# Logic-based assessment of the compatibility of UMLS ontology sources

**DOI:** 10.1186/2041-1480-2-S1-S2

**Published:** 2011-03-07

**Authors:** Ernesto Jiménez-Ruiz, Bernardo Cuenca Grau, Ian Horrocks, Rafael Berlanga

**Affiliations:** 1 Departamento de Lenguajes y Sistemas Informáticos, Universitat Jaume I, Campus de Riu Sec, Castellón, Spain; 2 Computing Laboratory, University of Oxford, Wolfson Building, Parks Road, Oxford, UK

## Abstract

**Background:**

The UMLS Metathesaurus (UMLS-Meta) is currently the most comprehensive effort for integrating independently-developed medical thesauri and ontologies. UMLS-Meta is being used in many applications, including* PubMed* and* ClinicalTrials.gov.* The integration of new sources combines automatic techniques, expert assessment, and auditing protocols. The automatic techniques currently in use, however, are mostly based on lexical algorithms and often disregard the semantics of the sources being integrated.

**Results:**

In this paper, we argue that UMLS-Meta’s current design and auditing methodologies could be significantly enhanced by taking into account the logic-based semantics of the ontology sources. We provide empirical evidence suggesting that UMLS-Meta in its 2009AA version contains a significant number of errors; these errors become immediately apparent if the rich semantics of the ontology sources is taken into account, manifesting themselves as unintended logical consequences that follow from the ontology sources together with the information in UMLS-Meta. We then propose general principles and specific logic-based techniques to effectively detect and repair such errors.

**Conclusions:**

Our results suggest that the methodologies employed in the design of UMLS-Meta are not only very costly in terms of human effort, but also error-prone. The techniques presented here can be useful for both reducing human effort in the design and maintenance of UMLS-Meta and improving the quality of its contents.

## Background

Ontologies — formal conceptualisations of a domain of interest in a machine-understandable format — are extensively used in bioinformatics. The most widely used ontology modelling language is the Web Ontology Language (OWL) [1] and its revision OWL 2 [2], which are World Wide Web Consortium (W3C) standards [3,4] The formal underpinning of OWL and OWL 2 is based on* formal logic *[5]. The key advantage of using logic over alternative representation mechanisms (e.g., semantic networks, frames, ER or UML diagrams) is that logic provides an unambiguous meaning to ontologies. As a result, ontologies can be used to process data (e.g., electronic patient records in the case of a medical application) in a more intelligent way. Prominent examples of biomedical OWL ontologies are the National Cancer Institute (NCI) Thesaurus [6,7], the Systematised Nomenclature of Medicine and Clinical Terms (SNOMED CT) [8], and the Foundational Model of Anatomy (FMA) [9]. These ontologies are gradually superseding the existing medical classifications and are becoming core platforms for accessing, gathering, and sharing biomedical knowledge and data.

SNOMED CT, NCI, and FMA describe partially overlapping domains. For example, both NCI and FMA describe the anatomy of the human heart; however, NCI describes the domain of human diseases, whereas FMA does not. Although the domains of interest of these ontologies may intuitively overlap, their vocabularies are most likely going to diverge. For example, NCI defines the entity “Myocardium” to describe the muscles that surround and power the human heart, whereas FMA uses the entity “Cardiac Muscle Tissue” to describe exactly the same set of muscles. This is because ontologies such as NCI and FMA have been independently developed and hence use different names and naming conventions for referring to their entities. Consequently, these ontologies, even if intuitively overlapping, are unrelated from a logical point of view. For example, if a data source described using FMA states that Mary Jones has suffered from an infarction affecting the “Cardiac Muscle Tissue”, and an NCI data source states that Paul Williams has suffered from an infarction affecting the “Myocardium” then a computer program would not be able to infer that both patients have suffered from the same condition.

To exchange or migrate data between ontology-based applications, it is crucial to establish correspondences (or* mappings*) between their ontologies. For example, a mapping between NCI and FMA should establish that the entities “Cardiac Muscle Tissue” and “Myocardium” are synonyms. Using this mapping, a computer program would then be able to migrate the data statement “Paul Williams has suffered from an infarction affecting the Myocardium” from an NCI-based application to an FMA-based application.

Ontology mappings are often conceptualised as tuples with the form 〈*id*, *e*_1_,* e*_2_*,n, p*〉 , where* id* is a unique identifier for the mapping,* e*_1_,* e*_2_ are entities in the vocabulary of the mapped ontologies, *n* is a numeric confidence measure between 0 and 1, and* ϱ* is a relation between *e*_1_ and * e*_2_,typically subsumption (i.e.,* e*_1_ is more specific than *e*_2_), equivalence (i.e., *e*_1_ and * e*_2_ are synonyms), or disjointness (i.e., no individual can be an instance of both *e*_1_ and * e*_2_) [10]. Creating such mappings manually is often unfeasible due to the size and complexity of modern biomedical ontologies. For example, SNOMED CT in its version from January 2009 contains more than 300,000 entities, while NCI (version 08.05d) and FMA (version 2.0) contain around 79,000 and 67,000 entities, respectively. Since the number of potential mappings grows (at least) quadratically with the number of entities in the relevant ontologies, a tool would need to consider (at least) 10 billion candidate mappings between SNOMED CT and NCI.

Most existing automated mapping generation techniques are based on* lexical* algorithms (e.g., [11-17]). Some of these algorithms are rather sophisticated and may exploit the syntactic structure of the ontologies [12,13] , or access external knowledge sources (e.g., WordNet [18]) to look for synonyms of ontology entities and their lexical variations [14,15,17].

The growing number of available techniques has made the creation of mappings between real-world ontologies possible. The most comprehensive effort for integrating biomedical ontologies through mappings is the UMLS Metathesaurus (UMLS-Meta) [19], which is being used in many applications, including *PubMed* (a search engine for accessing citations of biomedical articles) and* ClinicalTrials.gov* (a registry of clinical trials conducted around the world).

Currently, the integration of new ontologies in UMLS-Meta combines lexical algorithms, expert assessment [14,15,20,21], and auditing protocols [22]. In its 2009AA version, UMLS-Meta integrates more than one hundred thesauri and ontologies, including SNOMED CT, FMA, and NCI, and contains more than 6 million entities. UMLS-Meta provides a list with more than two million unique identifiers (CUIs). Each CUI can be associated to entities belonging to different sources. Pairs of entities from different sources with the same CUI are synonyms and hence can be represented as an equivalence mapping. It has been noticed that UMLS-Meta, despite being carefully curated by domain experts, may contain errors [23-27]. Current auditing techniques aimed at detecting potential errors mostly rely on UMLS-Meta* semantic network *[28] — a “top level” semantic model grouping the entities from the UMLS-Meta sources into suitable* semantic types.* Semantic types are then organised into so-called *semantic groups *[29]. For example “Heart” is associated with the semantic type “Body Part, Organ, or Organ Component” and the semantic group “Anatomy”. Errors can then be detected by identifying incompatibilities in the assignment of such semantic types to entities in the sources. For example the UMLS-Meta “Globular Actin” has, among others, “Amino Acid, Peptide, or Protein” and “Cell Component” as semantic types, which belong to two different semantic groups “Chemicals & Drugs” and “Anatomy” respectively.

In this paper, we argue that UMLS-Meta’s current design and auditing methodologies could be significantly enhanced by taking into account the logic-based semantics of the ontology sources. We provide empirical evidence suggesting that UMLS-Meta in its 2009AA version contains a significant number of errors; these errors become immediately apparent if the rich semantics of the ontology sources is taken into account, manifesting themselves as unintended logical consequences that follow from the ontology sources together with the information in UMLS-Meta. We then propose general principles and specific logic-based techniques to effectively detect and repair such errors. We have evaluated our techniques using three widely-used and rich ontology sources, namely FMA, NCI and SNOMED CT, and obtained very encouraging empirical results; our techniques, however, are generic and therefore applicable to any other UMLS-Meta source that can be expressed in OWL. Furthermore, we believe that our novel techniques are complementary to current UMLS-Meta auditing methods [22-26] and studying how they can be effectively combined constitutes an interesting direction for future work.

## Methods

The use of logic-based techniques requires the adoption of a* formal representation* of the mappings. This is crucial to reason unambiguously with the source ontologies and the corresponding mappings. Therefore, the first step in our research methodology has been to provide formal semantics to the mappings in UMLS-Meta. Once a coherent semantic framework has been identified, we can enable logical reasoning over the union of the source ontologies and their respective UMLS-Meta mappings and obtain logical consequences that were not derivable from any of them in isolation. Our main hypothesis is that such logical consequences can be used to identify errors in the mappings as well as to detect inherent incompatibilities between the source ontologies. To verify this hypothesis, we have identified three general principles, which describe precisely how these new logical consequences can be interpreted and exploited. Finally, we have designed a number of logic-based techniques that follow those general principles; these techniques are both semantically coherent and efficiently implementable in practice.

### Logical representation of ontology mappings

A number of formal representations for ontology mappings have been proposed so far in the literature (e.g., [10,30,31]), and there is currently no consensus on which ones are more suitable for practical applications. In this research, we have adopted a pragmatic approach in which a set of mappings is given as an OWL 2 ontology. Mappings 〈 *id, e*_1_*, e*_2_*, n, ϱ*〉are directly represented as OWL 2 axioms of the form SubClassOf(*e*_1_* e*_2_), EquivalentClasses(*e*_1_* e*_2_), or DisjointClasses(*e*_1_* e*_2_), for* ϱ* denoting subsumption, equivalence, and disjointness, respectively, and with* id* (the mapping id) and *n* (the confidence value) added as axiom annotations [2]. Such a representation seems semantically coherent, and allows us to reuse the extensive range of OWL-based algorithmic techniques and infrastructure that is currently available while preserving valuable information such as confidence values. In this setting, we can therefore restrict ourselves to consider the situation where OWL 2 ontologies *O*_1_ and *O*_2_ are integrated via a third OWL 2 ontology *M*, which contains the relevant mappings (e.g., *O*_1_ might be FMA, *O*_2_ NCI, and *M* UMLS-Meta). In the particular case of UMLS-Meta, we have processed the* MRCONSO* file from its distribution [32]. This file contains every entity in UMLS-Meta together with its* concept unique identifier* (CUI), its source vocabulary, its language, and other attributes not relevant for this work. Table 1 shows an excerpt from the rows in the* MRSCONSO* file associated to the CUI C0022417 (which represents the notion of “Joint”) with source vocabulary FMA, SNOMED CT or NCI.

**Table 1:** An excerpt from the MRCONSO file for “Joint”

CUI	Language	Source	Entity
		FMA	Joint
			
			Set_of_joints
		
C0022417	ENG	SNOMED CT	Joint_structure
		
		NCI	Joint
			
			Articulation

Table 1: An excerpt from the MRCONSO file for “Joint”

It follows from Table 1 that the notion of “Joint” is shared by FMA, SNOMED CT and NCI. In particular, FMA contains the entities* Joint* and* Set_of_joints,* NCI the entities* Articulation* and* Joint,* and SNOMED CT only the entity* Joint_structure*. All these entities have been annotated with the CUI C0022417 and therefore, according to UMLS-Meta’s intended meaning, they are synonyms. Then, for each pair of entities* e* and* e′* from* different* sources and annotated with the same CUI, we have generated the OWL 2 mapping axiom EquivalentClasses(*e e′*). The axioms obtained for our example CUI are given in Table 2. We do not explicitly generate axioms involving entities from the same source because we are interpreting UMLS-Meta as a mapping theory, whose purpose is to integrate independently developed sources, rather than to model the domain (i.e., to add explicit content to each of the sources independently). Note, however, that the mappings from Table 2 do modify the contents of the ontology sources* implicitly* (for example, they imply that the entities* Articulation* and* Joint* from NCI are equivalent, even if they are not in the original source). In the following section, we argue that such implicit modifications of a source due only to the mappings are one of the main causes of logical errors.

**Table 2:** Mappings between FMA, NCI and SNOMED CT

Mapped Ontologies	Generated Mappings
	EquivalentClasses(*FMA*:*Joint NCI*:*Joint*)
FMA ∼ NCI	EquivalentClasses(*FMA*:*Joint NCI*:*Articulation*)
	EquivalentClasses(*FMA*:*Set_of_joints NCI*:*Joint*)
	EquivalentClasses(*FMA*:*Set_of_joints NCI*:*Articulation*)

FMA ∼ SNOMED CT	EquivalentClasses(*FMA*:*Joint SNOMED*:*Joint_structure*)
	EquivalentClasses(*FMA*:*Set_of_joints SNOMED*:*Joint_structure*)

SNOMED CT ∼ NCI	EquivalentClasses(*SNOMED*:*Joint_ structure NCI*:*Joint*)
	EquivalentClasses(* SNOMED*:*Joint_structure NCI*:*Articulation*)

Table 2: Mappings between FMA, NCI and SNOMED CT. The prefixes “*FMA*:”, “*NCI*:” and “*SNOMED*:” are shown explicitly to emphasise that each ontology source uses a different namespace to refer to its entities. However, for simplicity, we will often obviate these prefixes in the text.

### Proposed principles

We have identified three general principles, which describe how logic-based techniques can be applied to the integration of two ontology sources *O*_1_ and *O*_2_ using a third mapping ontology *M*.

**1. The conservativity principle:** Given an ontology source (say, *O*_1_) and the mappings *M*, the union *O*_1_∪*M* should not introduce new semantic relationships between entities from *O*_1_.

**Figure 1:**
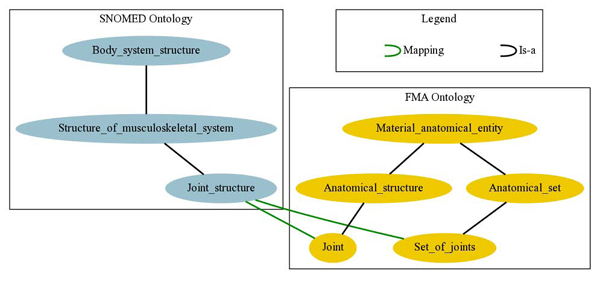
Conservativity principle violation between FMA and SNOMED CT mappings

The conservativity principle is based on the purpose of *M*, which is to enable the interaction between *O*_1_ and *O*_2_, rather than to provide a new description of the domain. In the case of our previous example about “Joints”, UMLS-Meta contains two mappings establishing the equivalence between the entity *Joint_structure* from SNOMED CT and the FMA entities* Joint* and* Set_of_joints* respectively. As a consequence, UMLS-Meta implies that* Joint* is also equivalent to* Set_of_joints.* However, in FMA* Joint* neither subsumes, nor it is subsumed by* Set_of_joints* (see Figure 1). The conservativity principle suggests that the obtained mappings are in conflict and (at least) one of them is likely to be incorrect.

**2. The consistency principle:** The ontology *O*_1_ ∪ *O*_2_ ∪*M* should be consistent and all the entities in its vocabulary should be satisfiable [5].

According to the consistency principle, the integration of well-established ontologies should not introduce logical inconsistencies, which are clear manifestations of a design error. These may be due to either erroneous mappings or to inherent incompatibilities between the source ontologies. In any case, in order for the integrated ontology to be successfully used in an application, these errors should be repaired by modifying either the source ontologies or the mappings.

**Figure 2:**
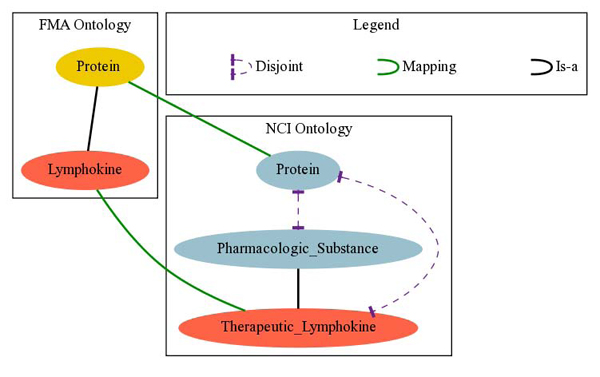
Consistency principle violation between FMA and NCI mappings

For example, as shown in Figure 2, UMLS-Meta maps the FMA concept* Protein* to the NCI concept *Protein,* and the FMA concept* Lymphokine* to the NCI concept* Therapeutic_Lymphokine.* In FMA, *Lymphokine* is a* Protein,* whereas in NCI* Therapeutίc_Lίmphokίne* is a* Pharmacologic_Substance.* Furthermore,* Pharmacologic_Substance* and* Protein* are disjoint in NCI and hence the union of NCI, FMA and UMLS-Meta would imply that* Lymphokine* and* Therapeutic_Limphokine* are unsatisfiable (i.e.,there can be no instances of either entity).

**3. The locality principle:** If two entities *e*_1_ and *e*_2_ from ontologies *O*_1_ and *O*_2_ are correctly mapped, then the entities semantically related to *e*_1_ in *O*_1_ are likely to be mapped to those semantically related to *e*_2_ in *O*_2_.

If the locality principle does not hold, then the following situations can be identified: (1) *M* may be incomplete and new mappings should be discovered, (2) the definitions of both concepts in their respective ontologies may be different or incompatible, or (3) the mapping between* e*_1_ and* e*_2_ may be erroneous.

### Specific techniques

We next propose a collection of logic-based techniques based on each of these general principles. Our techniques exploit the following observations about UMLS-Meta ontology sources and mappings:

● **Ob1:** The OWL 2 ontology *M* that encodes the contents of UMLS-Meta only contains axioms of the form EquivalentClasses(*e*_1_* e*_2_) where* e*_1_ is only mentioned in *O*_1_ and* e*_2_ is only mentioned in *O*_2_ (note that, as illustrated in Table 2, different ontology sources use different namespaces to refer to their entities). This observation is crucial to the design of techniques based on the conservativity principle.

● **Ob2:** UMLS-Meta ontology sources such as SNOMED CT, NCI and FMA contain both positive and negative information (e.g., if something is a “Protein”, then it is* not* a “Drug”). Logical inconsistencies can arise due to the simultaneous presence (either explicit or implicit) of two conflicting statements containing positive and negative information, e.g., the statement in FMA that lymphokine is a kind of protein and the (implicit) statement that lymphokine is not a kind of protein. This observation is important to design techniques based on the consistency principle.

● **Ob3:** The entities described in UMLS-Meta ontology sources such as SNOMED CT, NCI and FMA are “loosely interconnected”. Roughly speaking, this implies that the “meaning” of an entity in each of these ontologies only depends on a very small set of entities in the ontology that are “semantically related” to it. To formalise the notion of an entity being “semantically related” to another entity in an ontology, we use the logic-based ontology modularisation framework from [33]. This observation is important to design techniques based on either the consistency or the locality principles.

#### Techniques based on the conservativity principle

The conservativity principle can be directly expressed as the following* reasoning problem.* We say that an ontology source (say, *O*_1_) violates conservativity if there exists an OWL 2 axiom *α* such that *O*_1_ ∪*M* implies *α*, but *O*_1_ does not imply *α*. This problem is strongly related to the notion of* conservative extension *[34-36]. It is well-known that its computational complexity is very high even for lightweight ontology languages, and no practical algorithms currently exists.

As we describe next, however, in the case of UMLS-Meta we can exploit Observation **Ob1** to significantly simplify the problem.

Let *M* contain only axioms of the form EquivalentClasses(*e*_1_* e*_2_) where *e*_1_ is only mentioned in *O*_1_ and *e*_2_ is only mentioned in *O*_2_. Then, *O*_1_ violates conservativity if and only if there exist axioms EquivalentClasses(*e*_1_* e*_2_) and EquivalentClasses(*e′*_1_* e*_2_) in *M*, with *e*_1_ and *e′*_1_ different entities in *O*_1_, such that *O*_1_ alone does not imply the axiom EquivalentClasses(*e*_1_* e′*_1_).

If this is the case, then the mappings EquivalentClasses(*e*_1_* e*_2_) and EquivalentClasses(*e′*_1_* e*_2_) from *M* are in conflict and one of them may be incorrect. In our previous example (recall Figure 1), the mappings EquivalentClasses(* Joint_structure Joint)* and EquivalentClasses(* Joint_structure Set_of_joints)* between SNOMED CT and FMA are likely to be in conflict.

In order to identify such conflicting mappings, it suffices to (syntactically) check in *M* whether two entities from one of the sources (e.g.,* Joint* and* Set_of_ joints* from FMA) are mapped to the same entity in the other source (e.g.,* Joint_structure* from SNOMED CT) and then check (semantically) whether these two entities were already equivalent with respect (only) to the former source. These checks can be performed efficiently in practice: the former is syntactic, and the latter involves a single semantic test using an ontology reasoner (e.g., Does FMA imply that* Joint* and* Set_of_joints* are equivalent?).

#### Techniques based on the consistency principle

Similarly to the conservativity principle, the consistency principle can also be easily formulated as a reasoning problem. Let us denote with sig(*O*) the vocabulary of an ontology *O*. We say that *O*_1_, *O*_2_ and *M* violate consistency if there is an entity* e *∊ sig(*O*_1_) ∪ sig(*O*_2_) that is unsatisfiable with respect to *O*_1_ ∪*O*_2_ ∪ *M* (i.e., *O*_1_ ∪*O*_2_ ∪ *M* implies the axiom EquivalentClasses(*e owl:Nothing*)).

The obvious way to check consistency violation and identify sets of conflicting mappings is to use an ontology reasoner to check the satisfiability of each entity in the vocabulary of *O*_1_ ∪ *O*_2_ ∪ *M* and then apply state-of-the-art ontology debugging techniques to identify and disambiguate conflicts (e.g., see [30,37-41]). In our example from Figure 2, we could use a reasoner to identify that the concepts *Lymphokine* and* Therapeutic_Limphokine* are unsatisfiable with respect to the integration of FMA and NCI via UMLS-Meta. Then, debugging techniques could be used to identify the mappings and axioms from the source ontologies responsible for these errors.

This approach, however, can be computationally prohibitive; for example, the classification of the ontology obtained after integrating FMA and NCI using UMLS-Meta required almost 70 hours when using the reasoner HermiT [42] on a high performance server with 32GB of RAM; furthermore, the reasoner detected more than 15,000 unsatisfiable entities. Current debugging and repair techniques, which often rely on computing minimal sets of axioms responsible for each inconsistency, cannot be applied when the reasoning time is so prohibitive and the number of errors so high.

In our particular setting, unsatisfiable concepts can be caused by either erroneous mappings, or by inherent incompatibilities between the source ontologies. Our method relies on first identifying and disambiguating conflicting pairs of mappings (i.e., mappings that when occurring together make a concept unsatisfiable) and only then detecting and resolving incompatibilities between the sources.

**Detecting conflicting mappings.** We next exploit Observations **Ob2** and **Ob3** to define a simple heuristic technique, which is along the lines of those presented in [17,43,44].

The* disjointness-based inconsistency heuristic* is defined as follows. If* e* and* e′* from *O*_1_ are mapped to* f* and* f′* from *O*_2_ and *O*_1_ implies that* e* is subsumed by* e′*, but *O*_2_ implies that* f* and* f′* are disjoint, then the consistency principle is violated (recall Figure 2). Note that the converse does not necessarily hold. This heuristic requires semantic tests to check whether* e* is subsumed by* e′* in *O*_1_ and whether* f* and* f′* are disjoint in *O*_2_. However, this heuristic requires only the classification of each of the source ontologies *independently.* (Note that many reasoners can produce also the implicit disjointness relationships between their entities as an additional output of classification with only a relatively short additional delay.)

Furthermore, by Observation **Ob3** we can optimise even further and perform only the classification of the logic-based modules [33] for the mapped entities in each of the source ontologies. Consider, for example, the mappings between FMA and SNOMED CT. The logic-based module in FMA for the entities that are mapped to SNOMED CT contains only 10,204 entities (out of 67,000), and the corresponding module in SNOMED CT contains only 15,428 entities (out of 300,000). That is, extracting and classifying the modules instead of classifying the source ontologies as a whole results in a considerable simplification. Finally, the set of conflicting mappings is directly obtained and therefore there is no need to apply expensive debugging techniques to retrieve minimal sets of axioms responsible for the inconsistency.

**Figure 3:**
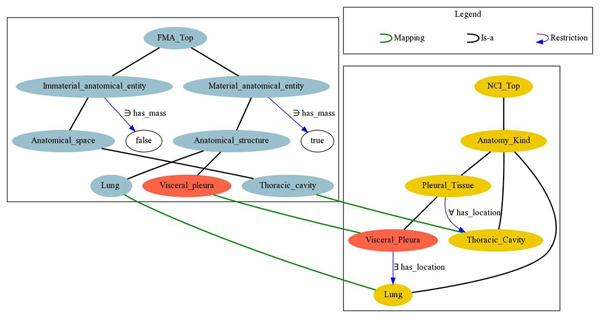
Unsatisfiability due to inherent incompatibilities between FMA and NCI

**Detecting incompatibilities between the sources.** Even if the mappings between two ontology sources are the intended ones, the sources may describe a particular aspect of the domain in incompatible ways. For example, consider Figure 3 describing the notion of “Visceral Pleura” in FMA and NCI. The three mappings between the entities “Visceral Pleura”, “Lung” and “Thoracic Cavity” in both ontologies are clearly the intended ones. However, their integration results in* Visceral_Pleura* becoming unsatisfiable. According to NCI, the visceral pleura is located in a lung; furthermore, it is a pleural tissue, which can only be located in the thoracic cavity. However, according to FMA the thoracic cavity is an immaterial anatomical entity, whereas the lung is a material anatomical entity. Finally, material and immaterial entities are disjoint, as implied by FMA. Therefore, the visceral pleura is located in some anatomical entity that is both material and immaterial, which leads to a contradiction.

We believe that, in such cases, the ontology engineer must participate in the repair process. In order to detect these incompatibilities and help the user to repair them, we provide tool support in our prototype ContentMap [30], which we briefly describe later on.

#### Techniques based on the locality principle

The conservativity and consistency principles allow us to identify pairs of mappings in UMLS-Meta that are in mutual conflict. However, since UMLS-Meta does not assign a confidence value to each mapping, it is not clear how to disambiguate these conflicts (e.g., how to decide whether to map* Joint_structure* to *Joint* or to* Set_of_joints).* If there are too many conflicts, manual disambiguation becomes unfeasible. We propose to apply the locality principle in order to compute a confidence value for each conflicting mapping, which we can then exploit for (partially) automating the disambiguation process. 

**Computing confidence values.** Assume that* e* from *O*_1_ is mapped via a mapping* μ* to* f* from *O*_2_. As already mentioned in Observation **Ob3**, the application of the locality principle relies on the well-known ontology modularisation framework from [33]. Therefore, if most of the entities in the module  for* e* in *O*_1_ (i.e., those entities that are “semantically related” to* e* in *O*_1_) are also mapped to those in the module  for* f* in *O*_2_, then we can assign a high confidence value to *μ*. Intuitively, such confidence values conf (*μ*) can be obtained by computing the ratio between the number of entities in the modules which are mapped via UMLS-Meta, and the total number of entities in the modules:

However, since the modules are of relatively small size, UMLS-Meta often does not contain enough mappings to obtain an accurate value. For example, UMLS-Meta maps* Upper_Extremity* from NCI and *Arm* from FMA, but none of the entities in the module for* Upper_Extremity* in NCI is mapped to an entity in the module for* Arm* in FMA. To address this issue, we use a lexical matching algorithm [11] to obtain additional lexical correspondences between entities in the modules and refine our confidence value.

**Figure 4:**
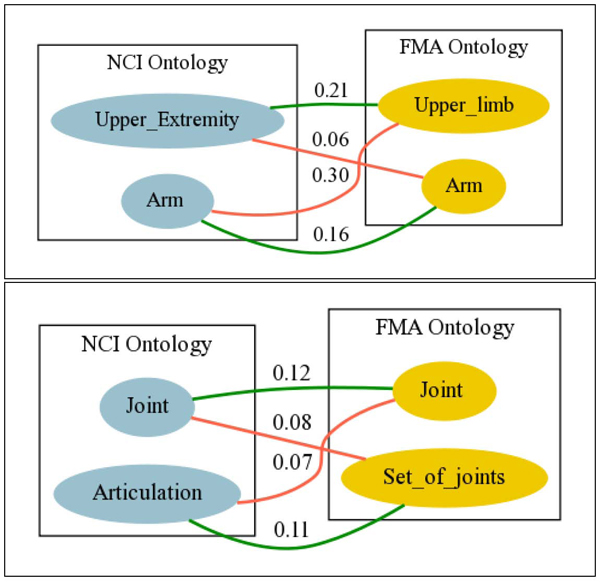
Combined cases of ambiguity

**Disambiguating conflicts automatically.** Figure 4 depicts sets of conflicting mappings obtained using the conservativity principle together with the confidence values we have obtained for each of them. Consider the mappings between NCI and FMA on the left-hand-side of the figure. Let *μ*_1_,* μ*_2_ represent the mappings respectively connecting* Upper_Extremity* in NCI to* Upper_limb* and* Arm* in FMA, and let *μ*_3_,*μ*_4_ represent those relating* Arm* in NCI to* Upper_Limb* and* Arm* from FMA, respectively. We can identify the following four conflicts:

*k*_1_ = {*μ*_1_, *μ*_2_}    *   k*_2_ = {*μ*_3_, *μ*_4_}    *   k*_3_ = {*μ*_1_, *μ*_3_}    *   k*_4_ = {*μ*_2_, *μ*_4_}

In order to disambiguate all the conflicts between two source ontologies, we need to remove one mapping per conflict in such a way that the result of adding their confidence values is minimised. This is a standard *diagnosis* problem, for which practical algorithms are well-known [45]. In our example, the solution involves removing the mappings *μ*_2_ (with confidence 0.06) and *μ*_3_ (with confidence 0.30).

#### Resolving inherent incompatibilities between source ontologies

Finally, as already mentioned, conflicts due to inherent incompatibilities between source ontologies (and not to unintended mappings) may require the intervention of a domain expert. However, as seen in Figure 3, source incompatibilities are not always apparent and therefore domain experts need to be supported by suitable tools. To provide the required tool support, we have developed the ContentMap prototype [30]. ContentMap helps users to understand the semantic consequences of the integration by identifying and explaining the new entailments that hold in the merged ontology, but not in *O*_1_, *O*_2_ and *M* alone.

Furthermore, ContentMap proposes different minimal repair plans for those entailments that the user indicates are unintended and ranks them accoding to the impact of their application. These plans may involve both the deletion or modification of source ontology axioms, and thus expert intervention is required to select the most appropriate repair.

## Implementation and empirical results

We have implemented our techniques and combined them in an integrated solution consisting of the following steps, which have been described in the Methods section:

1. *Mapping extraction:* given the ontology sources to be integrated, we extract the corresponding set of mappings expressed in OWL from UMLS-Meta’s MRCONSO file.

2. *Conflict detection*: given the OWL mappings previously extracted and the ontology sources in OWL format, we apply our techniques to compute sets of conflicting mappings.

3. *Automatic conflict disambiguation*: we apply our techniques based on the locality principle to compute a confidence value for each conflicting mapping; then, we apply repair methods to disambiguate each set of conflicting mappings.

4. *Semi-automatic repair of source incompatibilities*: we reason with the sources and the mappings resulting from automatic disambiguation, and then use ContentMap to detect and repair (with manual intervention) the remaining unintended entailments.

We have evaluated our techniques using UMLS-Meta version 2009AA and the corresponding versions of FMA, NCI and SNOMED CT, which contain 66,724, 78,989 and 304,802 entities, respectively. After translating the relevant parts of UMLS-Meta into OWL 2, we obtained 3,024 mapping axioms between FMA and NCI, 9,072 between FMA and SNOMED CT and 19,622 between SNOMED CT and NCI. The mapping extraction script from the MRCONSO file and the obtained OWL mappings are available online [46].

When reasoning over each of the source ontologies independently, all their entities were found satisfiable. However, after the respective integrations via UMLS-Meta mappings, we obtained a huge number of unsatisfiable entities, namely 5,015 when integrating FMA and NCI, 16,764 with FMA and SNOMED CT, and 76,025 with SNOMED CT and NCI. Thus, from a semantic point of view, the integration of these ontologies via UMLS-Meta is far from error-free.

In order to identify conflicts between the obtained UMLS-Meta mappings, we have then applied the conservativity and consistency principles. The conflict detection scripts, and the text files containing the obtained conflicts and the associated confidence values used for disambiguation can also be downloaded from [46].

● Using the principle of conservativity, we found 991 conflicting mapping between FMA and NCI, 2,426 between FMA and SNOMED CT and 9,080 between SNOMED CT and NCI.

● Using the disjointness-based inconsistency heuristic, we found 300 conflicting mapping pairs between FMA and NCI, 14,959 between FMA and SNOMED CT and 34,628 between SNOMED CT and NCI. Note that each of these conflicts will certainly lead to the unsatisfiability of an entity in the union of the respective source ontologies and UMLS-Meta mappings.

As discussed in the Methods section, the locality principle allows us to assign a confidence value to each conflicting mapping and then exploit this value to automatically disambiguate the identified conflicts. The automatic disambiguation process removed 570 (19%) of the mappings between FMA and NCI, 4,077 (45%) of those between FMA and SNOMED CT and 13,358 (63%) of those between SNOMED CT and NCI. The resulting disambiguated mappings are available online in OWL format (see Additional files 1, 2, 3) [46].

After automatic disambiguation, we found only 2 unsatisfiable entities when integrating FMA and NCI (recall example from Figure 3), 44 for FMA and SNOMED CT, and none for SNOMED CT and NCI. As already discussed, these errors are most likely due to inherent incompatibilities between the ontology sources; thus, expert assessment is required. Our tool ContentMap was then used to understand and repair these remaining inconsistencies. ContentMap proposed 19 repair plans for FMA and NCI and 372 FMA and SNOMED CT. We have inspected the top-ranked repair plans and found them intuitive and reasonable from a modelling perspective. For example, the repair plans for FMA and NCI suggested the deletion/modification of the NCI axiom

SubClassOf*(Pleural_Tissue* allValuesFrom (*has_location Thoracic_Cavity))* among others (recall example from Figure 3). A link to ContentMap’s binaries is provided in [46].

Despite the large number of deprecated mappings during automatic disambiguation, the integration of our source ontologies via UMLS-Meta still results in a considerable number of (possibly intended) new logical consequences. For example, after repairing all inconsistencies using ContentMap, the integration between FMA and SNOMED CT still results in 973 new subsumption relationships between FMA entities and 586 new subsumption relationships between SNOMED CT entities. Further manual revision (e.g., using again our tool ContentMap) would be necessary to determine which ones among these new consequences are deemed unintended.

## Discussion and conclusion

When integrating ontology sources via UMLS-Meta, the contents of the ontology sources should be taken into account. Several authors have proposed different kinds of* structural analysis* of SNOMED CT, FMA and NCI to evaluate their compatibility [47,48]. In this paper, we have argued that the rich* logic-based semantics* of these ontology sources should also be considered. Our specific contributions can be summarised as follows:

1. We have provided empirical evidence suggesting that, by taking into account the semantics of the sources, one can detect a significant number of additional errors in UMLS-Meta as well as many inherent incompatibilities between the sources’ description of particular domains.

2. We have proposed three general principles which describe how logic-based techniques can be applied to audit the integration of ontology sources. These principles are generic and could be applied to ontologies in any domain. The consistency principle has been used in a similar formulation in related literature and the principle of locality is (at least implicitly) behind the design of many structural heuristics for ontology matching. To the best of our knowledge, however, the principle of conservativity is novel and has not been used in the context of ontology mapping.

3. We have proposed and implemented logic-based techniques based on these principles. Those based on the conservativity and locality principles are novel with the latter exploiting state of the art ontology modularisation techniques.

4. We have adapted state of the art repair techniques to our setting by taking into account the confidence values obtained using the locality principle.

5. We have implemented an integrated solution that combines all our techniques in a systematic way, and evaluated it over UMLS-Meta with encouraging empirical results. Our techniques, however, could be used in contexts other than UMLS-Meta.

We consider that our results naturally complement those in [47,48], as well as current auditing methodologies in UMLS-Meta [22-26].

For future work, we plan to improve our techniques in several ways. First, our automatic disambiguation is rather aggressive, in the sense that a significant number of UMLS-Meta mappings are discarded in order to prevent logical errors. We plan to explore, on the one hand, how the disambiguation process can be relaxed to include as many of the original mappings in UMLS-Meta as possible and, on the other hand, how to improve the accuracy of the disambiguation confidence metrics based on the locality principle.

Second, our techniques rely on the information contained in UMLS-Meta’s MRCONSO file. UMLS-Meta, however, provides other sources of information. The semantic types and groups of UMLS-Meta could be given a logic-based interpretation (at least partially) and then used to enhance our heuristics.

Furthermore, the file AMBIGSUI.RRF, which contains a list of ambiguous and redundant terms, could be used for filtering out obvious logical errors.

Third, different ontology sources may describe the same concept at different levels of granularity, which may lead to incompatibilities when mapping those sources; this is a well-known (and open) issue in ontology matching. Although our logic-based heuristics can help identifying errors caused by such incompatibilities, they do not tackle this issue directly, which we leave for future research.

Finally, we aim at seeking feedback from domain experts concerning both the automatic and the tool-assisted disambiguation processes; this feedback could provide us with precision and recall values for our techniques.

## List of abbreviations

FMA: Foundational Model of Anatomy; NCI: National Cancer Institute; SNOMED CT: Systematised Nomenclature of Medicine and Clinical Terms; CUI: Concept Unique Identifier; OWL: Ontology Web Language; and UMLS: Unified Medical Language System

## Competing interests

The authors declare that they have no competing interests.

## Authors contributions

EJ and BCG conceived the project and led the development of the research. EJ also implemented the proposed techniques and performed their evaluation. IH and RB contributed to the discussions, supervised the progress of the project, and participated in the writing of the manuscript. All authors have read and approved the final manuscript.

## Supplementary Material

Additional file 1 Description of data: Disambiguated UMLS2009AA equivalence mappings (2454 from 3024) between FMA and NCI ontologies. File Format: OWL (Ontology Web Language) Click here for file

Additional file 2 Description of data: Disambiguated UMLS2009AA equivalence mappings (4995 from 9072) between FMA and SNOMED CT ontologies. File Format: OWL (Ontology Web Language) Click here for file

Additional file 3 Description of data: Disambiguated UMLS2009AA equivalence mappings (6264 from 19622) between NCI and SNOMED CT ontologies. File Format: OWL (Ontology Web Language) Click here for file
